# Non-invasive investigation of *Polychromophilus* parasite infections in bat populations in Serbia using bat flies

**DOI:** 10.1186/s13071-023-05786-1

**Published:** 2023-05-26

**Authors:** Branka Bajić, Oskar Werb, Ivana Budinski, Jelena Blagojević, Juliane Schaer, Jaap van Schaik

**Affiliations:** 1 Department of Genetic Research, Institute for Biological Research “Sinisa Stankovic”, National Institute of the Republic of Serbia, University of Belgrade, Belgrade, Serbia; 2 Department of Molecular Parasitology, Humboldt University, Berlin, Germany; 3 Department of Biological Sciences, Macquarie University, North Ryde, NSW Australia; 4 Department of Applied Zoology and Nature Conservation, University of Greifswald, Greifswald, Germany

**Keywords:** *Polychromophilus*, Nycteribiidae, *Miniopterus*, *Rhinolophus*, Ectoparasite, Haemosporida, Host specificity, Dipteran vector

## Abstract

**Background:**

Haemosporidian parasites of the genus *Polychromophilus* infect bats worldwide. They are vectored by obligate ectoparasitic bat flies of the family Nycteribiidae. Despite their global distribution, only five *Polychromophilus* morphospecies have been described to date. The two predominant species, *Polychromophilus melanipherus* and *Polychromophilus murinus*, are broadly distributed and mainly infect miniopterid and vespertilionid bats, respectively. In areas where species from different bat families aggregate together, the infection dynamics and ability of either *Polychromophilus* species to infect other host families is poorly characterized.

**Methods:**

We collected 215 bat flies from two bat species, *Miniopterus schreibersii* and *Rhinolophus ferrumequinum*, which sometimes form mixed clusters in Serbia. *Miniopterus schreibersii* is known to be frequently infected with *P. melanipherus*, whereas *R. ferrumequinum* has been observed to be incidentally infected with both *Polychromophilus* species. All flies were screened for *Polychromophilus* infections using a PCR targeting the haemosporidian *cytb* gene. Positive samples were subsequently sequenced for 579 bp of cytochrome b (*cytb*) and 945 bp of cytochrome oxidase subunit 1 (*cox1*).

**Results:**

*Polychromophilus melanipherus* DNA was detected at six out of nine sampling locations and in all three examined bat fly species collected from *M. schreibersii* (*Nycteribia schmidlii*, *n* = 21; *Penicillidia conspicua*, *n* = 8; *Penicillidia dufourii*, *n* = 3). Four and five haplotypes were found for *cytb* and *cox1*, respectively. Evidence for multiple *Polychromophilus* haplotypes was found in 15 individual flies. These results point to a high diversity of *P. melanipherus* parasites in *Miniopterus* hosts and efficient transmission throughout the study area. A single *Phthiridium biarticulatum* bat fly collected from *R. ferrumequinum* screened positive for *P. melanipherus*, but only yielded a partial *cox1* sequence fragment. Nevertheless, this result suggests that secondary hosts (both bat and fly species) are regularly confronted with this parasite.

**Conclusions:**

The results of this study provide new insights into the prevalence and distribution of *Polychromophilus* parasites in European bats and their nycteribiid vectors. The use of bat flies for the non-invasive investigation of *Polychromophilus* infections in bat populations has proven to be efficient and thus represents an alternative for large-scale studies of infections in bat populations without the need to invasively collect blood from bats.

**Graphical Abstract:**

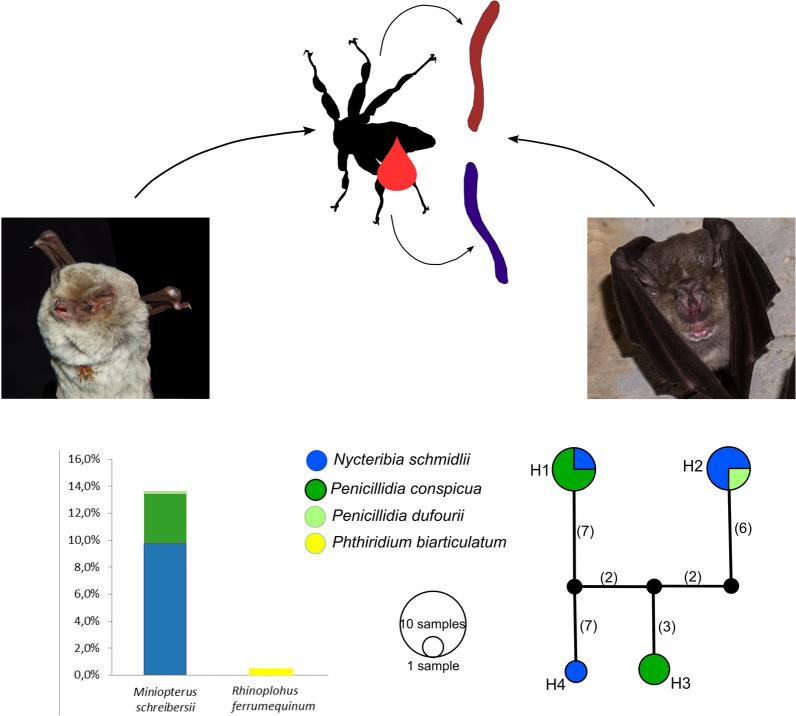

**Supplementary Information:**

The online version contains supplementary material available at 10.1186/s13071-023-05786-1.

## Background

The order Haemosporida comprises over 500 species from at least 15 described genera, which infect a wide range of vertebrates and are transmitted by several families of haematophagous dipterans [1, 2]. The genus *Polychromophilus* is one of nine haemosporidian genera that infect bats [3], but is the only genus with a global distribution, which also includes the temperate zones. Of the five described species of *Polychromophilus*, two have been reported in Europe: *Polychromophilus melanipherus*, which is mainly found in miniopterid bat species [3–7], and *P. murinus*, which primarily infects a range of vespertilionid bat species [8–11]. However, both species have been reported to infect bat species from other families, including rhinolophids [7, 9]. A single case of simultaneous infection with both species, based on morphology, was reported from a *Rhinolophus ferrumequinum* in Italy [9]. Overall, patterns of host specificity and exposure of bat individuals to both parasite species remain poorly understood.

*Polychromophilus* parasites are vectored by nycteribiid flies, which are wingless, haematophagous, obligate ectoparasites of bats, and live within the pelage of their hosts [12, 13]. *Polychromophilus* gametocytes (the vertebrate blood stages of the parasite) are ingested by the bat flies during a blood meal from an infected bat host. After sexual development in the midgut of the bat fly, the sporozoite stages migrate to the salivary glands of the fly and are transferred to the bat during the next blood meal [8, 9].

Adult bat flies are unable to disperse more than a few meters independently and transmission occurs mainly by direct physical contact between bats. As fly offspring pupate on roost walls, transmission may also take place through successive use of the same roost sites by different host species without direct physical contact. Most nycteribiid fly species are highly host-specific [14, 15], even when offered a chance to switch between them [16]. Nevertheless, occurrences on secondary hosts are occasionally observed [17]. Therefore, spillover infections of *Polychromophilus* parasites from individuals of one bat species to another could occur if multiple bat species cluster together or roost in close proximity within a roost.

Natural caves and other large underground roosts often provide shelter for more than one species of cave-dwelling bats, which in some cases may form mixed-species clusters [18, 19]. In Europe, the single representative of the bat family Miniopteridae, *Miniopterus schreibersii*, forms large colonies, often in association with other cave-dwelling bat species of other bat families, such as the vespertilionid species *Myotis myotis*, *M. blythii*, *Myotis capaccinii* [20] and the rhinolophid species *R. ferrumequinum* [16]. *Miniopterus schreibersii* is considered a regional migrant, usually changing roosts within 100 km between seasons [21, 22], and rarely undertaking longer migrations [23]. In contrast to *M. schreibersii*, the colonies of *R. ferrumequinum* are smaller and the distances between summer and winter roosts are shorter [23, 24]. *Rhinolophus ferrumequinum* often forms monotypic colonies, but may occasionally mix with *Myotis emarginatus*, *Rhinolophus euryale*, *R. blasii* and *M. schreibersii* [16, 25]. In Serbia, several roosts are known where both *M. schreibersii* and *R. ferrumequinum* are present and sometimes form mixed clusters as well as sites where one of the two species is absent [25].

In this study, we examined *Polychromophilus* parasite infections in multiple species of nycteribiid bat flies collected from *M. schreibersii* and *R. ferrumequinum* in Serbia and Bosnia and Herzegovina. We aimed to characterize the prevalence and genetic diversity of *Polychromophilus* infections in this system. Molecular screening of flies provides an efficient method to quantify the opportunity and frequency of parasite transmission, without the need for more invasive blood sampling in bats [6]. European *Rhinolophus* species have occasionally been found to be infected with *P. murinus,* with infections documented in single individuals of *Rhinolophus* sp. from Bulgaria [26] and *Rhinolophus hipposideros*, *R. ferrumequinum* and *R. mehelyi* in Bulgaria/Romania [7]. We investigated the presence of *Polychromophilus* parasites in flies collected from *R. ferrumequinum* to explore whether infections occur in this host species in shared roosts with *Miniopterus* bat hosts or whether the infections are present independently in *R. ferrumequinum*. Since it is likely that the two focal host bat species are infected with the two different haemosporidian species *P. melanipherus* and *P. murinus*, we defined spillover as infection of the fly species with the *Polychromophilus* species not normally associated with the bat host from which it was collected. We hypothesized that, despite the high host specificity of the fly species previously observed in this system (see Pejić et al. [16]), even accidental spillover would be sufficient to introduce both *Polychromophilus* species into each host/fly system, and allow their persistence, when both bat species are present.

## Methods

### Sampling

**Fig. 1:**
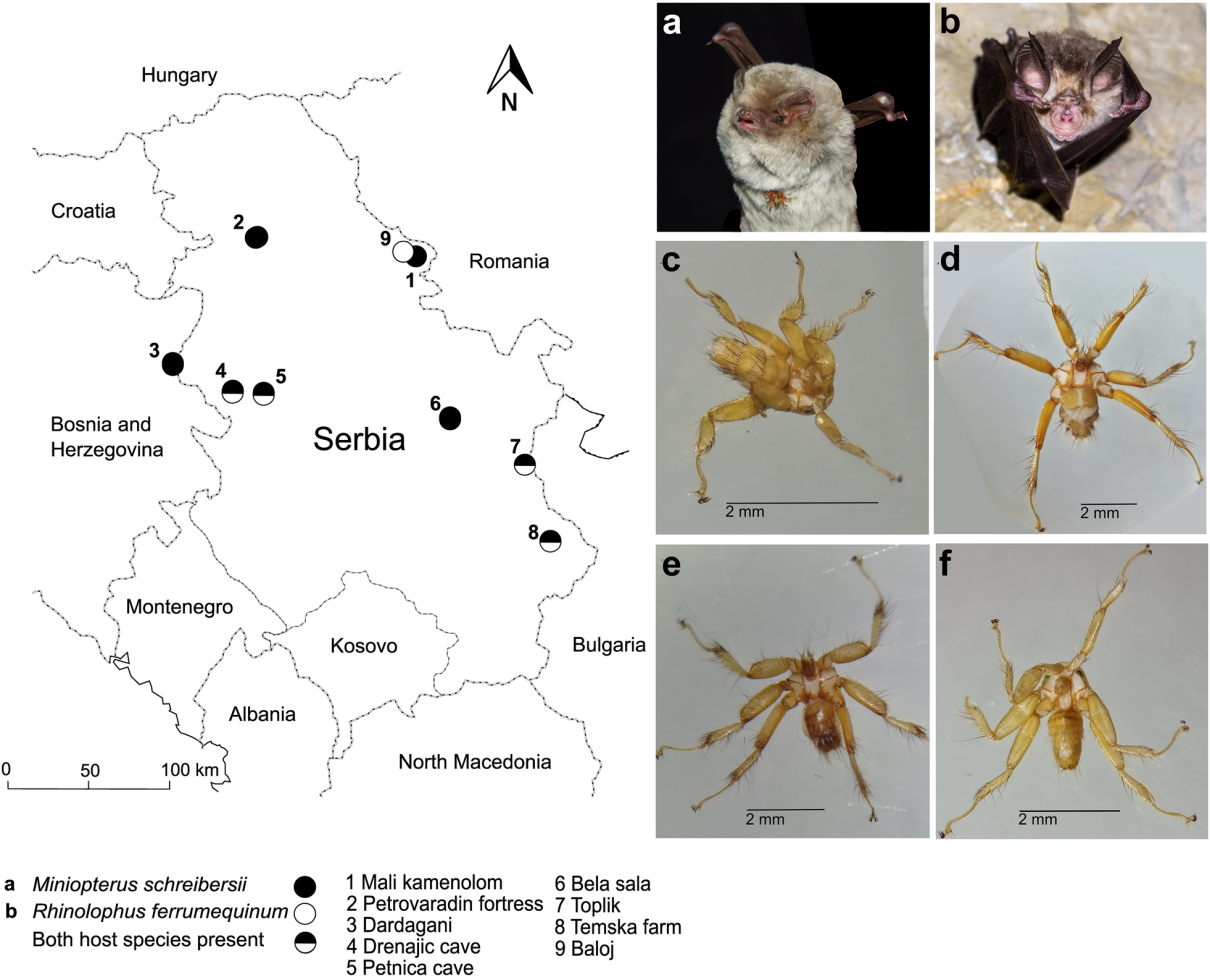
Map of sampling sites in Serbia and Bosnia and Herzegovina. Insets: **a** *Miniopterus schreibersii*; **b** *Rhinolophus ferrumequinum*; **c** *Nycteribia schmidlii*; **d** *Penicillidia conspicua*; **e** *Penicillidia dufourii;* **f** *Phthiridium biarticulatum*

Nine different roosting sites were sampled, eight in Serbia and one in Bosnia and Herzegovina [16], of which four were shared by the bat species *M. schreibersii* and *R. ferrumequinum*, four were exclusively used by *M. schreibersii* and one by *R. ferrumequinum* (Fig. 1). Sampling was conducted during the summer and autumn seasons in 2017 and 2018. Bats were captured using mist nets or by hand net inside the roosts and released immediately after processing. Bat flies were collected using fine-toothed forceps and stored individually in 99% ethanol. Bat flies were identified to species level both morphologically (after Theodor [27]) and genetically [16]. A total of 215 bat flies (150 from *M. schreibersii* and 65 from *R. ferrumequinum*) were examined for the presence of *Polychromophilus* parasite DNA. The flies of *M. schreibersii* belonged to the three bat fly species *Nycteribia schmidlii* (*n* = 133), *Penicillidia conspicua* (*n* = 14) and *Penicillidia dufourii* (*n* = 3). All bat flies from *R. ferrumequinum* were identified as *Phthiridium biarticulatum* (*n* = 65) [16].

### Molecular methods

DNA was extracted from the entire fly specimens using the Mag-Bind Blood and Tissue DNA HDQ extraction kit (Omega) according to the manufacturer’s protocols. The extracted bat fly DNA was screened for the presence of *Polychromophilus* parasite DNA, which could be present in either the parasitic vector stages and/or the blood meal content (which includes the *Polychromophilus* gametocyte blood stages from the vertebrate host) of the bat fly. An initial screening PCR targeted approximately 600 bp of the haemosporidian mitochondrial cytochrome b (hereafter *cytb*) gene using the primer combination 3932F and DW4 [28]. PCR was performed using the QIAGEN TopTaq Master Mix or the QIAGEN AllTaq Master Mix Kit with 2–4 μl of genomic DNA as template and 1 μl of each primer (10 mM) in a total volume of 20 µl. For samples with confirmed *Polychromophilus* infections (i.e. successful amplification using the 3932F-DW4 primer pair), we sequenced both the amplified fragment of *cytb* as well as a 950-bp fragment of cytochrome oxidase 1 (hereafter *cox1*) following established protocols (Additional file 1: Table S1) [28, 29]. At least two independent amplification attempts were performed per sample, and a positive control was included in all PCRs.

### DNA sequencing and phylogenetic analysis

Nucleotide sequences were edited using Geneious Prime 2021.1 (https://www.geneious.com). Individual sequence assemblies were manually checked, and sequences were compared to resolve ambiguous base calls. Double nucleotide peaks (≥ 40% height at one nucleotide position) in the sequence electropherograms of high-quality sequence segments in individual sequence assemblies were recorded as mixed haplotype infection. Double peaks generally aligned with polymorphic sites, and the two peaks corresponded to the two bases observed in other haplotypes; therefore, we excluded the possibility of a sequencing error. Ambiguous base calls were coded with the corresponding IUPAC ambiguity code, and missing data were coded as N. Sequences were subsequently aligned using the Muscle algorithm [30] implemented in Geneious Prime 2021.1 and trimmed to obtain uniform sequence lengths (579 bp for *cytb*, 945 bp for *cox1*). Sequences were compared to reference sequences of *Polychromophilus murinus* and *P. melanipherus* on GenBank (https://www.ncbi.nlm.nih.gov/genbank/) to confirm species identity.

Sequences without ambiguous bases were used to construct haplotype networks for *cytb* (*n* = 13), *cox1* (*n* = 17) and a concatenation of both fragments (*n* = 11). Median-joining haplotype networks were constructed in PopART v.1.7 [31] and labeled according to the bat fly species from which the *Polychromophilus* sequence was amplified. Finally, we compared the haplotypes obtained in this study to all existing *P. melanipherus* sequences on GenBank [4–7, 32–40] for both *cytb* and *cox1* (Additional file 1: Table S2). Both sequences were trimmed to improve overlap with existing sequences (*cytb*: 479 bp, *n* = 119; *cox1*: 768 bp, *n* = 51). Median-joining haplotype networks were constructed in PopART v.1.7 [31] and labeled according to the country or region of origin.

## Results

### Prevalence

*Polychromophilus* DNA was detected in 33 of the 215 screened bat flies (15%), including individuals of all four bat fly species examined and both bat host species (Table 1). Infections were recorded at six out of nine sampling locations (Table 1). Overall prevalence was low (< 5 infections per site), with the exception of Dardagani, where 17 out of 20 bat flies were found to be infected. Nearly all *Polychromophilus* infections were detected in bat flies collected from *M. schreibersii*, with only one infection detected in a bat fly collected from *R. ferrumequinum*.

**Table 1:** Bat flies positive to presence of *Polychromophilus* DNA, by species and sampling sites, including summaries of the number of mixed infections, and the overall prevalence per site and per fly species

Host species	*Miniopterus schreibersii*			*Rhinolophus ferrumequinum*		
Bat fly species	*Nycteribia schmidlii*	*Penicillidia conspicua*	*Penicillidia dufourii*	*Phthiridium biarticulatum*	Mixed infection	Overall prevalence
1 Mali kamenolom	1/20	–	–	–	1/1	1/20
2 Petrovaradin fortress	1/9	3/5	–	–	0/4^a^	4/14
3 Dardagani	10/13	4/4	3/3	–	11/17	17/20
4 Drenajicka cave	0/16	0/2		0/4	–	0/22
5 Petnica cave	5/16	1/3	–	0/7	2/5	5/26
6 Bela sala	0/20	–	–	–	–	0/20
7 Toplik	1/20	–	–	1^b^/20	1/2^a^	2/40
8 Temska farm	4/19	–	–	0/19	0/4	4/38
9 Baloj	–	–	–	0/15	–	0/15
Total per species	21/133	8/14	3/3	1/65	15/33	33/215

^a^One sample per site could not be amplified with sufficient quality to determine if the infection was composed of multiple haplotypes

^b^The single *Ph. biarticulatum* sample screened positive for *Polychromophilus* for both *cytb* and *cox1*, but sequencing of the PCR products was only successful for the *cox1* sequence

### Genetic diversity

All 33 parasite infections were identified as *P. melanipherus* parasites based on their *cytb* and/or *cox1* nucleotide sequence identities with reference sequences in NCBI. Remarkably, a high proportion of the positive samples (15/33, Table 1) exhibited infections with multiple haplotypes, visible as a double nucleotide peak in at least one base in either *cytb* or *cox1* sequences (Additional file 1: Table S3).

**Fig. 2:**
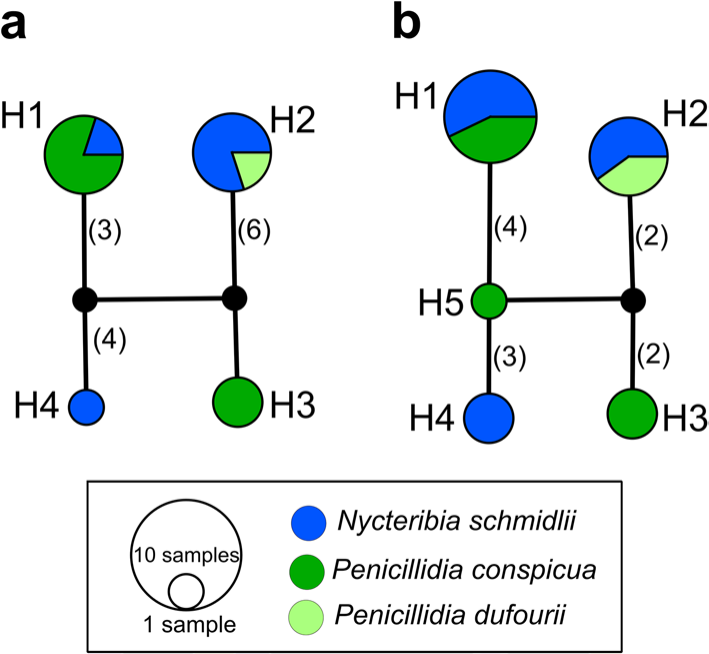
Haplotype network analysis of *Polychromophilus melanipherus* parasites in the different bat fly species. **a** *cytb* (579 bp) *n* = 13; **b** *cox1* (945 bp) *n* = 17. The line between haplotypes or nodes represents one base change, unless otherwise labeled in parenthesis

For *cytb* (579 bp), 13 samples could be unambiguously aligned, yielding four haplotypes (Fig. 2a). For *cox1* (945 bp), five haplotypes were found across the 17 samples without ambiguous sites (Fig. 2b). The topology of both networks is identical, with the exception of the additional fifth haplotype in *cox1*. The haplotype network of the concatenated dataset of samples where both *cytb* and *cox1* nucleotide sequences were available without ambiguities (11/33) was composed of four haplotypes (note: no *cytb* sequence was available for the single sample with *cox1*-H5) (Additional file 1: Fig. S1).

Haplotypes were not structured according to location, and no consistent clustering of haplotypes according to bat fly species was observed. We were only able to obtain a single short *cox1*-sequence (432 bp, albeit of high quality) for the single *Polychromophilus* infection detected in a bat fly collected from *R. ferrumequinum*. This sequence represented a mixed infection of haplotypes H1 and H4 and is thus not represented in the haplotype networks.

**Fig. 3:**
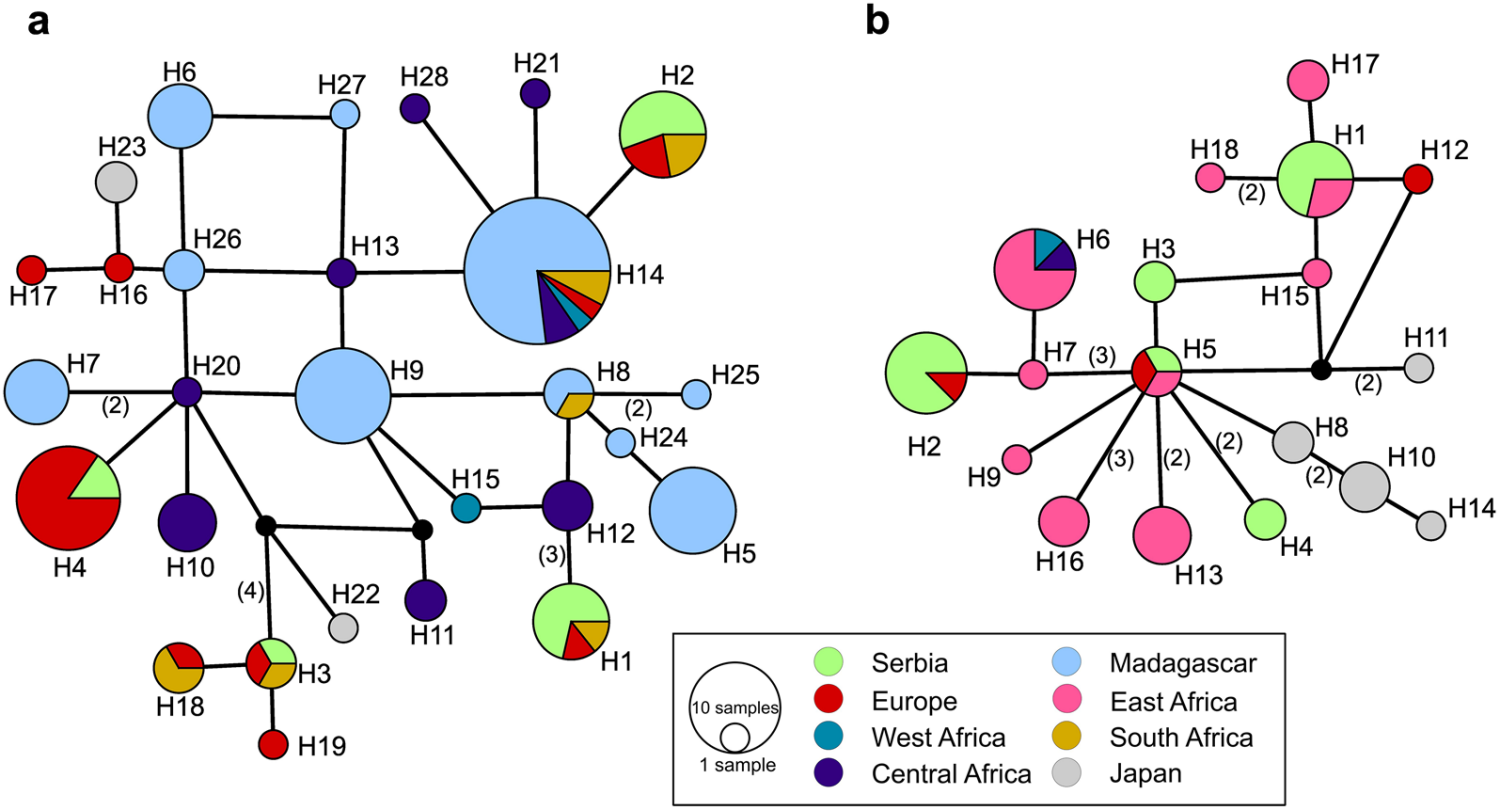
Haplotype network analysis of *Polychromophilus melanipherus* around the world **a** for the gene *cytb* (479 bp) *n* = 119; **b** for the gene *cox1* (768 bp), *n* = 51. The line between haplotypes or nodes represents one base change, unless otherwise labeled in parentheses

All four *cytb* haplotypes aligned with 100% pairwise identity to previously published haplotypes (Fig. 3a). All have been previously observed in Europe, and H1–H3 have also been reported from South Africa (Additional file 1: Table S2). Two of the five *cox1* haplotypes (H2, H5; Fig. 3b) similarly shared 100% identity with published *P. melanipherus* haplotypes from Europe (Additional file 1: Table S2) and two shared identity with haplotypes from East Africa (H1, H5). The remaining *cox1* haplotypes (H3 and H4) were not previously reported.

## Discussion

In a sample of bat flies from two bat hosts that frequently share roosts and occasionally form mixed clusters in Serbia and Bosnia and Herzegovina, we detected the DNA of *P. melanipherus* in 15% of the 215 bat flies examined. Positives were found in all four sampled fly species. All but one of the positive detections were found in bat flies collected from the bat *M. schreibersii*. *Polychromophilus* infections were present at six out of nine sampling sites in this study, and sequencing revealed that nearly half (15/33) of all positive samples represented mixed infections with multiple *P. melanipherus* haplotypes. Thus, in line with surveys from surrounding countries [e.g. 6, 7], our results suggest that *M. schreibersii* bats and their associated nycteribiid flies are frequent hosts of *P. melanipherus* in Serbia and Bosnia and Herzegovina.

We found a markedly higher prevalence in the two investigated *Penicillidia* species (*P. conspicua* 57%; *P. dufourii* 100%; *N. schmidlii* 15.8%), although sample sizes were lower. Being nearly twice as large, we speculate that *Penicillidia* fly species may exhibit higher infection rates because they either take larger blood meals or feed more frequently. This is particularly notable as both *Penicillidia* species are oligoxenous and frequently found on other cave-roosting species in the area [17, 41, 42]. *Penicillidia dufourii* has previously also been found to be infected with *P. murinus* in samples collected from *Myotis* bat hosts [7]. Taken together, the high prevalence of *Polychromophilus* in these fly species, their ability to vector both European *Polychromophilus* species and their broad bat host range perfectly exemplify the potential for both *Polychromophilus* species to spill over in cave roosts with mixed bat assemblages.

Despite this potential, our data suggest that *Polychromophilus* infections in *R. ferrumequinum* bats and their flies are rare in our study sites, as we detected *Polychromophilus* DNA only in a single *Ph. biarticulatum* bat fly. The single recovered *cox1 Polychromophilus* sequence originated from Toplik and represents a mixed haplotype infection of two *P. melanipherus* haplotypes (H1 + H4), which were also found in bat flies from *M. schreibersii* in this study. This represents the first record of *P. melanipherus* DNA from *Ph. biarticulatum*, which nearly exclusively parasitizes rhinolophid bats (as was also observed in this study system; [16]). In contrast, the other *Polychromophilus* species present in Europe, *P. murinus*, has been found in three rhinolophid bat species, including *R. ferrumequinum* [7], but no infections were detected in the current study.

Overall, considering the intricate roost sharing and the formation of mixed clusters between *R. ferrumequinum* and *M. schreibersii*, as well as other *Myotis* bats, we posit that there might be barriers that limit the persistence of *Polychromophilus* infections in *R. ferrumequinum* and/or *Ph. biarticulatum*. Whether this barrier is caused by the incompatibility of the bat host or the reduced suitability of its bat flies as a vector remains to be investigated. For example, the single positive *Ph. biarticulatum* observed here might stem from an infected *R. ferrumequinum* bat or could represent a fly that recently took a blood meal from an infected *M. schreibersii* bat and subsequently transferred to a *R. ferrumequinum* host. In either case, the detection of *Polychromophilus* in the bat fly sample could indicate a true infection of the bat fly (i.e. where the *Polychromophilus* parasite successfully completes its sexual development cycle in the fly host) or only be present in the most recent blood meal that the fly took. Thus, the infection source and ability of this fly species to vector the parasite cannot be confirmed.

The 15 samples that yielded a mixed-haplotype infection may be the result of a bat fly feeding on two different bat individuals that harbored different *Polychromophilus* haplotypes. Coincidentally, most mixed haplotypes were retrieved from sampling sites with high prevalence of *Polychromophilus* infections. Mixed haplotypes have only been described twice before for *Polychromophilus* parasite infections [7, 43], while mixed haplotype infections are common in *Hepatocystis*, another haemosporidian taxon that infects bats (among other mammals) [43–45]. However, *Hepatocystis* parasites are transmitted by *Culicoides* species (Ceratopogonidae), temporarily haematophagous ectoparasites [1]. The high proportion of mixed haplotype infections in our sample size suggests that each of these bat individuals was repeatedly infected with different *Polychromophilus* haplotypes transmitted by different fly individuals. Alternatively, each fly individual could have fed on different infected bat individuals (that featured different *Polychromophilus* haplotypes) and developed a mixed infection in the process. To answer this question, future studies could investigate the *Polychromophilus* infection in both the blood sample of the bat and its corresponding bat fly and compare the *Polychromophilus* haplotypes. In general, *Polychromophilus* haplotype analysis will help understanding bat/bat fly interactions and transmission between bats through the contact among bats from different roosts.

At a broader scale, our genetic results support the notion that *P. melanipherus* is effectively dispersed over large distances across the range of its Miniopterid bat hosts [40]. The four *cytb* and five *cox1* unambiguously aligned haplotypes of *P. melanipherus* recovered here were genetically diverse, each being separated by multiple polymorphisms. The four recovered *cytb* haplotypes corresponded to sequences previously recovered elsewhere in Europe or South Africa. For *cox1*, three of the five haplotypes similarly corresponded to published haplotypes from Europe or East Africa. Indeed, in a haplotype network of all available sequence data, the haplotypes recovered in this study were distributed throughout the overall network. This suggests either a comparatively recent colonization of European populations or an ongoing transmission across *Miniopterus* bat hosts throughout the Eastern Hemisphere.

## Conclusions

The results of this study provide new insights into the prevalence, distribution and genetic diversity of *Polychromophilus* parasites in European bats and their nycteribiid vectors. We report a single case of spillover of *P. melanipherus* infection in a fly (*Ph. biarticulatum*) collected from a *R. ferrumequinum* bat host. The use of bat flies for the non-invasive study of *Polychromophilus* infections in bat populations has proven to be very efficient (see also [6, 7]) and thus represents an alternative for large-scale investigations of infections in bat populations without the need to invasively collect blood from bats.

## Supplementary Information

**Additional file 1. Table S1**: Primer names, sequences and sources of protocols used for their amplification; **Table S2**: GenBank accession numbers of previously published sequences of *cytb* and *cox1* genes of *Polychromophilus melanipherus*; **Table S3**: Overview of *Polychromophilus melanipherus cytb* and *cox1* haplotypes obtained; **Figure S1**: Haplotype network of concatenated sequences of genes *cytb* and *cox1* of *Polychromophilus melanipherus* 1524 bp long.

## Data Availability

Nucleotide sequences generated during this study were deposited in GenBank under accession nos. OQ357633—OQ357642. Further information regarding all positive samples is provided in Additional file 1: Table S3.
