# Supplementary material for: Non-invasive investigation of Polychromophilus parasite infections in bat populations in Serbia using bat flies
Source: Parasit Vectors. 2023 May 26;16:170. doi: 10.1186/s13071-023-05786-1 (PMC10214537; doi:10.1186/s13071-023-05786-1)
Supplement: Supplementary file 1 — Additional file 1. Table S1: Primer names, sequences and sources of protocols used for their amplification; Table S2: GenBank accession numbers of previously published sequences of cytb and cox1 genes of Polychromophilus melanipherus; Table S3: Overview of Polychromophilus melanipherus cytb and cox1 haplotypes obtained; Figure S1: Haplotype network of concatenated sequences of genes cytb and cox1 of Polychromophilus melanipherus 1524 bp long. [file 13071_2023_5786_MOESM1_ESM.pdf]

Additional file 1

Supplementary material to: B. Bajić, O Werb, I. Budinski, J. Blagojević, J Schaer, J. van Schaik (2023) Non-invasive investigation of *Polychromophilus* parasite infections in bat populations in Serbia using bat flies, submitted to Parasites & Vectors

Corresponding authors:

Jaap van Schaik: vanschaika@uni-greifswald.de

Juliane Schaer: schaerju@hu-berlin.de

Table of contents

Table S1 – page 1

Table S2 – page 2

Table S3 – page 9

Figure S1 – page 11

References – page 12

Table S1: Primer names, sequences and sources of protocols used for their amplification.

Gene	Primer	Sequence (5'->3')	Reference
**cytochrome b**	3932F	GGGTTATGTATTACCTTGGGGTC	Perkins & Schall 2002
(*cytb*)	DW4	TGTTTGCTTGGGAGCTGTAATCATAATGTG	Perkins & Schall 2002
**cytochrome c oxidase 1**	Cox1-R-out	AGGAATACGTCTAGGCATTACATTAAATCC	Martinsen et al. 2008
(*cox1*)	Cox1-R-in	GTATTTTCTCGTAATGTTTTACCAAAGAA	Martinsen et al. 2008
	Cox1-F-out	CTATTTATGGTTTTCATTTTTATTTGGTA	Martinsen et al. 2008
	Cox1-F-in	ATGATATTTACARTTCAYGGWATTATTATG	Martinsen et al. 2008
	Cox1-mid-F	TTATTCTGGTTTTTTGGTCATCCAG	Martinsen et al. 2008
	Cox1-mid-R	CTGGATGACCAAAAAACCAGAATAA	Martinsen et al. 2008

Table S2: GenBank accession numbers of previously published sequences

Supplementary table of GenBank accession numbers of sequences of cytchrome b (cytb) and cytochrome oxidase subunit 1 (cox1) of *Polychromophilus melanipherus* parasite, from other authors. Haplotype codes correspond to Figure 3 of the study. Sequences with [a] correspond to haplotypes found in this study.

No.	GenBank acc.no.	Author	Host species	Gene	Origin locality	Locality name given here	Haplotype code given here
1	AY762067	Duval et al. 2004 Unpublished	-	cytb	Madagascar	Madagascar	H6
2	AY762068	Duval et al. 2004 Unpublished	-	cytb	Madagascar	Madagascar	H6
3	AY762069	Duval et al. 2004 Unpublished	-	cytb	Madagascar	Madagascar	H7
4	AY762070	(Duval et al. 2007)	*Miniopterus manavi*	cytb	Madagascar	Madagascar	H8
5	AY762071	(Duval et al. 2007)	-	cytb	Madagascar	Madagascar	H9
6	AY762074	(Duval et al. 2007)	-	cytb	Madagascar	Madagascar	H5
7	JQ995284	(Duval et al. 2012)	*Miniopterus inflatus*	cytb	Gabon	Central Africa	H10
8	JQ995285	(Duval et al. 2012)	*Miniopterus inflatus*	cytb	Gabon	Central Africa	H11
9	JQ995286	(Duval et al. 2012)	*Miniopterus inflatus*	cytb	Gabon	Central Africa	H12
10	JQ995287	(Duval et al. 2012)	*Miniopterus inflatus*	cytb	Gabon	Central Africa	H13
11	JQ995288	(Duval et al. 2012)	*Miniopterus inflatus*	cytb	Gabon	Central Africa	H14
12	KF159681	(Schaer et al. 2013)	*Miniopterus villiersi*	cytb	Guinea	West Africa	H15
13	KF159699	(Schaer et al. 2013)	*Miniopterus villiersi*	cytb	Guinea	West Africa	H14
14	KJ131270[a]	(Witsenburg et al. 2015)	*Miniopterus schreibersii*	cytb	Croatia, Portugal, Spain, Switzerland, Italy, Slovakia, France	Europe	H2[a]

15	KJ131271	(Witsenburg et al. 2015)	*Miniopterus schreibersii*	cytb	Croatia, Portugal, Spain, Switzerland, Italy, Slovakia, France	Europe	H16
16	KJ131272[a]	(Witsenburg et al. 2015)	*Miniopterus schreibersii*	cytb	Croatia, Portugal, Spain, Switzerland, Italy, Slovakia, France	Europe	H3[a]
17	KJ131273[a]	(Witsenburg et al. 2015)	*Miniopterus schreibersii*	cytb	Croatia, Portugal, Spain, Switzerland, Italy, Slovakia, France	Europe	H1[a]
18	KJ131274	(Witsenburg et al. 2015)	*Miniopterus schreibersii*	cytb	Croatia, Portugal, Spain, Switzerland, Italy, Slovakia, France	Europe	H17
19	KJ131275[a]	(Witsenburg et al. 2015)	*Miniopterus schreibersii*	cytb	Croatia, Portugal, Spain, Switzerland, Italy, Slovakia, France	Europe	H4[a]
20	KJ131276	(Witsenburg et al. 2015)	*Miniopterus schreibersii*	cytb	Croatia, Portugal, Spain, Switzerland, Italy, Slovakia, France	Europe	H18
21	KJ131277	(Witsenburg et al. 2015)	*Miniopterus schreibersii*	cytb	Croatia, Portugal, Spain, Switzerland, Italy, Slovakia, France	Europe	H19
22	KU182361	(Obame-Nkoghe et al. 2016)	*Nycteribia schmidlii*	cytb	Gabon	Central Africa	H10

23	KU182362	(Obame-Nkoghe et al. 2016)	*Nycteribia schmidlii*	cytb	Gabon	Central Africa	H10
24	KU182363	(Obame-Nkoghe et al. 2016)	*Nycteribia schmidlii*	cytb	Gabon	Central Africa	H20
25	KU182364	(Obame-Nkoghe et al. 2016)	*Nycteribia schmidlii scotti*	cytb	Gabon	Central Africa	H12
26	KU182365	(Obame-Nkoghe et al. 2016)	*Nycteribia schmidlii scotti*	cytb	Gabon	Central Africa	H12
27	KU182366	(Obame-Nkoghe et al. 2016)	*Nycteribia schmidlii*	cytb	Gabon	Central Africa	H11
28	KU182367	(Obame-Nkoghe et al. 2016)	*Nycteribia schmidlii*	cytb	Gabon	Central Africa	H21
29	KU182368	(Obame-Nkoghe et al. 2016)	*Penicillidia fulvida*	cytb	Gabon	Central Africa	H14
30	LC668428	(Rosyadi et al. 2022)	*Miniopterus fuliginosus*	cytb	Japan	Japan	H22
31	LC668429	(Rosyadi et al. 2022)	*Miniopterus fuliginosus*	cytb	Japan	Japan	H23
32	LC668430	(Rosyadi et al. 2022)	*Miniopterus fuliginosus*	cytb	Japan	Japan	H23
33	MH744503	(Ramasindrazana et al. 2018)	*Miniopterus mahafaliensis*	cytb	Madagascar	Madagascar	H5
34	MH744504	(Ramasindrazana et al. 2018)	*Miniopterus mahafaliensis*	cytb	Madagascar	Madagascar	H5
35	MH744505	(Ramasindrazana et al. 2018)	*Miniopterus mahafaliensis*	cytb	Madagascar	Madagascar	H5
36	MH744506	(Ramasindrazana et al. 2018)	*Miniopterus griffithsi*	cytb	Madagascar	Madagascar	H5
37	MH744507	(Ramasindrazana et al. 2018)	*Miniopterus mahafaliensis*	cytb	Madagascar	Madagascar	H8
38	MH744508	(Ramasindrazana et al. 2018)	*Miniopterus griveaudi*	cytb	Madagascar	Madagascar	H24
39	MH744509	(Ramasindrazana et al. 2018)	*Miniopterus gleni*	cytb	Madagascar	Madagascar	H9
40	MH744511	(Ramasindrazana et al. 2018)	*Miniopterus gleni*	cytb	Madagascar	Madagascar	H25
41	MH744512	(Ramasindrazana et al. 2018)	*Miniopterus manavi sensu lato*	cytb	Madagascar	Madagascar	H9
42	MH744513	(Ramasindrazana et al. 2018)	*Miniopterus manavi sensu lato*	cytb	Madagascar	Madagascar	H9
43	MH744514	(Ramasindrazana et al. 2018)	*Miniopterus griveaudi*	cytb	Madagascar	Madagascar	H9
44	MH744515	(Ramasindrazana et al. 2018)	*Miniopterus griveaudi*	cytb	Madagascar	Madagascar	H9
45	MH744517	(Ramasindrazana et al. 2018)	*Miniopterus*	cytb	Madagascar	Madagascar	H9

			griveaudi				
46	MH744518	(Ramasindrazana et al. 2018)	*Miniopterus gleni*	cytb	Madagascar	Madagascar	H14
47	MH744519	(Ramasindrazana et al. 2018)	*Miniopterus griffithsi*	cytb	Madagascar	Madagascar	H14
48	MH744520	(Ramasindrazana et al. 2018)	*Paratriaenops furculus*	cytb	Madagascar	Madagascar	H26
49	MH744521	(Ramasindrazana et al. 2018)	*Miniopterus gleni*	cytb	Madagascar	Madagascar	H26
50	MH744522	(Ramasindrazana et al. 2018)	*Miniopterus griveaudi*	cytb	Madagascar	Madagascar	H6
51	MH744523	(Ramasindrazana et al. 2018)	*Miniopterus griveaudi*	cytb	Madagascar	Madagascar	H6
52	MH744524	(Ramasindrazana et al. 2018)	*Miniopterus griveaudi*	cytb	Madagascar	Madagascar	H27
53	MH744525	(Ramasindrazana et al. 2018)	*Miniopterus griveaudi*	cytb	Madagascar	Madagascar	H6
54	MH744526	(Ramasindrazana et al. 2018)	*Miniopterus manavi sensu lato*	cytb	Madagascar	Madagascar	H7
55	MH744527	(Ramasindrazana et al. 2018)	*Nycteribia stylidiopsis*	cytb	Madagascar	Madagascar	H5
56	MH744528	(Ramasindrazana et al. 2018)	*Penicillidia leptothrinax*	cytb	Madagascar	Madagascar	H7
57	MH744529	(Ramasindrazana et al. 2018)	*Penicillidia leptothrinax*	cytb	Madagascar	Madagascar	H9
58	MH744530	(Ramasindrazana et al. 2018)	*Penicillidia leptothrinax*	cytb	Madagascar	Madagascar	H9
59	MH744531	(Ramasindrazana et al. 2018)	*Penicillidia leptothrinax*	cytb	Madagascar	Madagascar	H9
60	MK098848	(Rosskopf et al. 2018)	*Miniopterus minor*	cytb	Gabon	Central Africa	H10
61	MK098849	(Rosskopf et al. 2018)	*Miniopterus minor*	cytb	Gabon	Central Africa	H28
62	MT996236[a]	(Sándor et al. 2021)	*Miniopterus schreibersi*	cytb	Romania	Europe	H2[a]
63	MT996237[a]	(Sándor et al. 2021)	*Miniopterus schreibersi*	cytb	Romania	Europe	H4[a]
64	MW007671[a]	(Szentiványi et al. 2020)	*Nycteribia schmidlii*	cytb	South Africa	South Africa	H3[a]
65	MW007672	(Szentiványi et al. 2020)	*Nycteribia schmidlii*	cytb	South Africa	South Africa	H18
66	MW007673	(Szentiványi et al. 2020)	*Nycteribia schmidlii*	cytb	South Africa	South Africa	H18

67	MW007674[a]	(Szentiványi et al. 2020)	*Nycteribia schmidlii*	cytb	South Africa	South Africa	H2[a]
68	MW007675[a]	(Szentiványi et al. 2020)	*Nycteribia schmidlii*	cytb	South Africa	South Africa	H2[a]
69	MW007676[a]	(Szentiványi et al. 2020)	*Nycteribia schmidlii*	cytb	South Africa	South Africa	H1[a]
70	MW007677	(Szentiványi et al. 2020)	*Miniopterus natalensis*	cytb	South Africa	South Africa	H8
71	MW007678	(Szentiványi et al. 2020)	*Miniopterus natalensis*	cytb	South Africa	South Africa	H14
72	MW007679	(Szentiványi et al. 2020)	*Miniopterus natalensis*	cytb	South Africa	South Africa	H14
73	MW007680[a]	(Szentiványi et al. 2020)	*Nycteribia schmidlii*	cytb	Hungary	Europe	H4[a]
74	MW007681[a]	(Szentiványi et al. 2020)	*Nycteribia schmidlii*	cytb	Hungary	Europe	H4[a]
75	MW007682[a]	(Szentiványi et al. 2020)	*Nycteribia schmidlii*	cytb	Italy	Europe	H4[a]
76	MW007683[a]	(Szentiványi et al. 2020)	*Nycteribia schmidlii*	cytb	Italy	Europe	H4[a]
77	MW007684[a]	(Szentiványi et al. 2020)	*Nycteribia schmidlii*	cytb	Italy	Europe	H4[a]
78	MW007685	(Szentiványi et al. 2020)	*Nycteribia schmidlii*	cytb	Spain	Europe	H14
79	MW007686[a]	(Szentiványi et al. 2020)	*Miniopterus schreibersi*	cytb	Hungary	Europe	H4[a]
80	MW007687[a]	(Szentiványi et al. 2020)	*Miniopterus schreibersi*	cytb	Italy	Europe	H4[a]
81	MW007688[a]	(Szentiványi et al. 2020)	*Miniopterus schreibersi*	cytb	Italy	Europe	H4[a]
82	MW007689[a]	(Szentiványi et al. 2020)	*Miniopterus schreibersi*	cytb	Spain	Europe	H4[a]
83	MW039207	(Rasoanoro et al. 2021)	*Miniopterus sp.*	cytb	Madagascar	Madagascar	H9
84	MW039208	(Rasoanoro et al. 2021)	*Miniopterus griveaudi*	cytb	Madagascar	Madagascar	H14
85	MW039209	(Rasoanoro et al. 2021)	*Miniopterus ambohitrensis*	cytb	Madagascar	Madagascar	H14
86	MW039210	(Rasoanoro et al. 2021)	*Miniopterus gleni*	cytb	Madagascar	Madagascar	H14
87	MW039211	(Rasoanoro et al. 2021)	*Miniopterus gleni*	cytb	Madagascar	Madagascar	H14
88	MW039212	(Rasoanoro et al. 2021)	*Miniopterus gleni*	cytb	Madagascar	Madagascar	H14
89	MW039213	(Rasoanoro et al. 2021)	*Miniopterus gleni*	cytb	Madagascar	Madagascar	H14

90	MW039214	(Rasoanoro et al. 2021)	*Miniopterus gleni*	cytb	Madagascar	Madagascar	H14
91	MW039215	(Rasoanoro et al. 2021)	*Miniopterus gleni*	cytb	Madagascar	Madagascar	H14
92	MW039216	(Rasoanoro et al. 2021)	*Miniopterus gleni*	cytb	Madagascar	Madagascar	H14
93	MW039217	(Rasoanoro et al. 2021)	*Miniopterus gleni*	cytb	Madagascar	Madagascar	H14
94	MW039218	(Rasoanoro et al. 2021)	*Miniopterus gleni*	cytb	Madagascar	Madagascar	H14
95	MW039219	(Rasoanoro et al. 2021)	*Miniopterus gleni*	cytb	Madagascar	Madagascar	H14
96	MW039220	(Rasoanoro et al. 2021)	*Miniopterus gleni*	cytb	Madagascar	Madagascar	H14
97	MW039221	(Rasoanoro et al. 2021)	*Miniopterus gleni*	cytb	Madagascar	Madagascar	H14
98	MW039222	(Rasoanoro et al. 2021)	*Miniopterus gleni*	cytb	Madagaskar	Madagascar	H14
99	MW039223	(Rasoanoro et al. 2021)	*Miniopterus gleni*	cytb	Madagaskar	Madagascar	H14
100	MW039224	(Rasoanoro et al. 2021)	*Miniopterus gleni*	cytb	Madagaskar	Madagascar	H14
101	MW039225	(Rasoanoro et al. 2021)	*Miniopterus gleni*	cytb	Madagaskar	Madagascar	H14
102	MW039226	(Rasoanoro et al. 2021)	*Miniopterus ambohitrensis*	cytb	Madagaskar	Madagascar	H5
103	MW039227	(Rasoanoro et al. 2021)	*Miniopterus gleni*	cytb	Madagaskar	Madagascar	H5
104	MW039228	(Rasoanoro et al. 2021)	*Miniopterus gleni*	cytb	Madagaskar	Madagascar	H5
105	MW039229	(Rasoanoro et al. 2021)	*Miniopterus griveaudi*	cytb	Madagaskar	Madagascar	H7
106	MW039230	(Rasoanoro et al. 2021)	*Miniopterus ambohitrensis*	cytb	Madagascar	Madagascar	H7
1	JN990714	(Witsenburg et al. 2012)	*Miniopterus schreibersii*	cox1	Switzerland	Europe	H12
2	JN990716[a]	(Witsenburg et al. 2012)	*Miniopterus schreibersii*	cox1	Switzerland	Europe	H2[a]
3	JN990717[a]	(Witsenburg et al. 2012)	*Miniopterus schreibersii*	cox1	Switzerland	Europe	H5[a]
4	MK098851	(Rosskopf et al. 2018)	*Miniopterus minor*	cox1	Gabon	Central Africa	H6
5	KF159795	(Schaer et al. 2013)	*Miniopterus villiersi*	cox1	Guinea	West Africa	H6
6	KT750434	(Lutz et al. 2016)	*Miniopterus sp.*	cox1	Tanzania	East Africa	H6
7	KT750435	(Lutz et al. 2016)	*Miniopterus sp.*	cox1	Tanzania	East Africa	H16
8	KT750437	(Lutz et al. 2016)	*Miniopterus rufus*	cox1	Kenya	East Africa	H6
9	KT750438	(Lutz et al. 2016)	*Miniopterus rufus*	cox1	Kenya	East Africa	H9
10	KT750440	(Lutz et al. 2016)	*Miniopterus rufus*	cox1	Kenya	East Africa	H6
11	KT750441	(Lutz et al. 2016)	*Miniopterus rufus*	cox1	Kenya	East Africa	H15

12	KT750442[a]	(Lutz et al. 2016)	*Miniopterus rufus*	cox1	Kenya	East Africa	H5[a]
13	KT750443	(Lutz et al. 2016)	*Miniopterus rufus*	cox1	Kenya	East Africa	H6
14	KT750444	(Lutz et al. 2016)	*Miniopterus rufus*	cox1	Kenija	East Africa	H6
15	KT750445	(Lutz et al. 2016)	*Miniopterus rufus*	cox1	Kenya	East Africa	H6
16	KT750448	(Lutz et al. 2016)	*Miniopterus natalensis*	cox1	Kenya	East Africa	H13
17	KT750452[a]	(Lutz et al. 2016)	*Miniopterus natalensis*	cox1	Kenya	East Africa	H1[a]
18	KT750453	(Lutz et al. 2016)	*Miniopterus natalensis*	cox1	Kenya	East Africa	H7
19	LC715187	(Rosyadi et al. 2022)	*Miniopterus fuliginosus*	cox1	Japan	Japan	H8
20	LC715188	(Rosyadi et al. 2022)	*Miniopterus fuliginosus*	cox1	Japan	Japan	H8
21	LC715189	(Rosyadi et al. 2022)	*Miniopterus fuliginosus*	cox1	Japan	Japan	H11
22	LC715190	(Rosyadi et al. 2022)	*Miniopterus fuliginosus*	cox1	Japan	Japan	H10
23	LC715191	(Rosyadi et al. 2022)	*Miniopterus fuliginosus*	cox1	Japan	Japan	H14
24	LC715192	(Rosyadi et al. 2022)	*Miniopterus fuliginosus*	cox1	Japan	Japan	H10
25	LC715193	(Rosyadi et al. 2022)	*Miniopterus fuliginosus*	cox1	Japan	Japan	H10
26	KT750433	(Lutz et al. 2016)	*Miniopterus sp.*	cox1	Tanzania	East Africa	H17
27	KT750439	(Lutz et al. 2016)	*Miniopterus rufus*	cox1	Kenya	East Africa	H16
28	KT750447	(Lutz et al. 2016)	*Miniopterus natalensis*	cox1	Kenya	East Africa	H13
29	KT750449	(Lutz et al. 2016)	*Miniopterus natalensis*	cox1	Kenya	East Africa	H16
30	KT750450[a]	(Lutz et al. 2016)	*Miniopterus natalensis*	cox1	Kenya	East Africa	H1[a]
31	KT750451	(Lutz et al. 2016)	*Miniopterus natalensis*	cox1	Kenya	East Africa	H13
32	KT750541	(Lutz et al. 2016)	*Miniopterus sp.*	cox1	Tanzania	East Africa	H18
33	KT750455	(Lutz et al. 2016)	*Miniopterus natalensis*	cox1	Kenya	East Africa	H17
34	KT750456	(Lutz et al. 2016)	*Miniopterus natalensis*	cox1	Kenya	East Africa	H13

Table S3: Overview of *Polychromophilus melanipherus* cytb and cox1 haplotypes obtained per individual in this study.

**Sample No**	**Bat fly species**	**Bat host species**	**locality**	**Cytb haplotypes (H1-H4)**	**Cox1 haplotypes (H1-H5)**
A03865-2	*P. biarticulatum*	*R. ferrumequinum*	Toplik	NA	mixed
B04674-1	*N. schmidlii*	*M. schreibersii*	Temska farm	NA	**H2§**
B05253-2	*N. schmidlii*	*M. schreibersii*	Petnica cave	NA	mixed
B05253-3	*N. schmidlii*	*M. schreibersii*	Petnica cave	NA	mixed
B05257-1	*N. schmidlii*	*M. schreibersii*	Petnica cave	NA	**H3**
B05258-1	*N. schmidlii*	*M. schreibersii*	Petnica cave	**H2**	**H2**
B05261-1	*P. conspicua*	*M. schreibersii*	Petnica cave	**H2**	**H2**
B05422-1	*N. schmidlii*	*M. schreibersii*	Toplik	NA	§§
B05847-2	*N. schmidlii*	*M. schreibersii*	Mali kamenolom	mixed	NA
B06009-1	*P. conspicua*	*M. schreibersii*	Petrovaradin fortress	**H2**	**H2**
B06035-1	*N. schmidlii*	*M. schreibersii*	Dardagani	**H1**	**H1**
B06037-1	*N. schmidlii*	*M. schreibersii*	Dardagani	mixed	mixed
B06037-2	*N. schmidlii*	*M. schreibersii*	Dardagani	mixed	mixed
B06037-3	*N. schmidlii*	*M. schreibersii*	Dardagani	mixed	mixed
B06038-1	*P. conspicua*	*M. schreibersii*	Dardagani	**H2**	**H2**
B06038-2	*P. conspicua*	*M. schreibersii*	Dardagani	mixed	mixed
B06038-3	*P. conspicua*	*M. schreibersii*	Dardagani	mixed	**H5**
B06038-4	*N. schmidlii*	*M. schreibersii*	Dardagani	mixed	mixed
B06040-1	*P. dufourii*	*M. schreibersii*	Dardagani	mixed	mixed
B06040-3	*P. conspicua*	*M. schreibersii*	Dardagani	**H2**	mixed
B06041-1	*N. schmidlii*	*M. schreibersii*	Dardagani	**H1**	mixed
B06042-1	*N. schmidlii*	*M. schreibersii*	Dardagani	**H3**	**H3**
B06045-1	*N. schmidlii*	*M. schreibersii*	Dardagani	mixed	mixed
B06047-1	*P. dufourii*	*M. schreibersii*	Dardagani	**H1**	**H1**
B06047-2	*N. schmidlii*	*M. schreibersii*	Dardagani	**H1**	**H1**
B06047-3	*P. dufourii*	*M. schreibersii*	Dardagani	mixed	**H1**
B06047-4	*N. schmidlii*	*M. schreibersii*	Dardagani	**H1**	**H1**
B06063-4	*N. schmidlii*	*M. schreibersii*	Temska farm	NA	**H2**
B06063-8	*N. schmidlii*	*M. schreibersii*	Temska farm	NA	**H2**

B06063-9	*N. schmidlii*	*M. schreibersii*	Temska farm	NA	**H2**
B06130-1	*N. schmidlii*	*M. schreibersii*	Petrovaradin fortress	NA	§§
B08165-1	*P. conspicua*	*M. schreibersii*	Petrovaradin fortress	**H4**	**H4**
B08165-4	*P. conspicua*	*M. schreibersii*	Petrovaradin fortress	**H4**	**H4**

§Short sequence, §§ Sequence quality too bad for analysis, but BLASTn search identified *P. melanipherus*, NA = no sequence could be generated

All haplotypes can be accessed on GenBank under the following accession numbers:

Haplotype	Accession Number
Cytb-H1	OQ357633
Cytb-H2	OQ357634
Cytb-H3	OQ357635
Cytb-H4	OQ357636
Cox1-H1	OQ357637
Cox1-H2	OQ357638
Cox1-H3	OQ357639
Cox1-H4	OQ357640
Cox1-H5	OQ357641
Cox1-mixed	OQ357642

Figure S1: Concatenated haplotype network of *Polychromophilus melanipherus* cytb (579 bp) and cox1 (945 bp) sequence fragments for all 11 samples where both fragments were available without ambiguities.

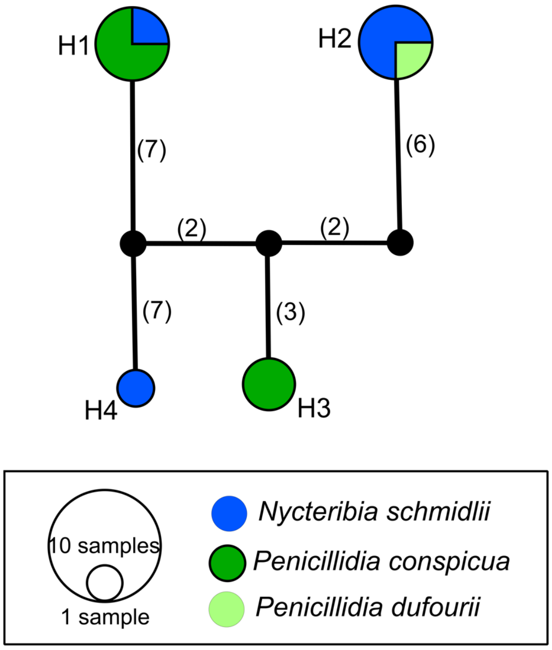

References

Duval L, Robert V, Csorba G, Hassanin A, Randrianarivelojosia M, Walston J, et al. Multiple host-switching of Haemosporidia parasites in bats. Malar J. 2007;6:1–8.

Duval L, Mejean C, Maganga GD, Makanga BK, Mangama Koumba LB, Peirce MA, et al. The chiropteran haemosporidian *Polychromophilus melanipherus*: A worldwide species complex restricted to the family Miniopteridae. Infect Genet Evol. 2012;12:1558–66.

Lutz HL, Patterson BD, Kerbis Peterhans JC, Stanley WT, Webala PW, Gnoske TP, et al. Diverse sampling of East African haemosporidians reveals chiropteran origin of malaria parasites in primates and rodents. Mol Phylogenet Evol. 2016;99:7–15.

Martinsen ES, Perkins SL, Schall JJ. A three-genome phylogeny of malaria parasites (*Plasmodium* and closely related genera): evolution of life-history traits and host switches. Mol Phylogenet Evol. 2008;47:261–73.

Obame-Nkoghe J, Rahola N, Bourgarel M, Yangari P, Prugnolle F, Maganga GD, et al. Bat flies (Diptera: Nycteribiidae and Streblidae) infesting cave-dwelling bats in Gabon: Diversity, dynamics and potential role in *Polychromophilus melanipherus* transmission. Parasites & Vectors. 2016;9:1–12.

Perkins S, Schall JJ. A molecular phylogeny of malarial parasites recovered from cytochrome b gene sequences. J Parasitol. 2002;88:972–8.

Ramasindrazana B, Goodman SM, Dsouli N, Gomard Y, Lagadec E, Randrianarivelojosia M, et al. *Polychromophilus* spp. (Haemosporida) in Malagasy bats: Host specificity and insights on invertebrate vectors. Malar J. 2018;17:1–11.

Rasoanoro M, Goodman SM, Randrianarivelojosia M, Rakotondratsimba M, Dellagi K, Tortosa P, et al. Diversity, distribution, and drivers of *Polychromophilus* infection in Malagasy bats. Malar J. 2021;20:1–11.

Rosskopf SP, Held J, Gmeiner M, Mordmüller B, Matsiégui PB, Eckerle I, et al. *Nycteria* and *Polychromophilus* parasite infections of bats in Central Gabon. Infect Genet Evol. 2018;68:30–4.

Rosyadi I, Shimoda H, Takano A, Yanagida T, Sato H. Isolation and molecular characterization of *Polychromophilus* spp. (Haemosporida: Plasmodiidae) from the Asian long - fingered bat (*Miniopterus fuliginosus*) and Japanese large - footed bat (*Myotis macrodactylus*) in Japan. Parasitol Res. 2022; 121(9):2547–2559.

Sándor AD, Péter Á, Corduneanu A, Barti L, Csősz I, Kalmár Z, et al. Wide distribution and diversity of malaria-related haemosporidian parasites (*Polychromophilus* spp.) in bats and their ectoparasites in Eastern Europe. Microorganisms. 2021;9:1–12.

Schaer J, Perkins SL, Decher J, Leendertz FH, Fahr J, Weber N, et al. High diversity of West African bat malaria parasites and a tight link with rodent *Plasmodium* taxa. Proc Natl Acad Sci. 2013;110:17415–9.

Szentiványi T, Markotter W, Dietrich M, Clément L, Ançay L, Brun L, et al. Host conservation through their parasites: Molecular surveillance of vector-borne microorganisms in bats using ectoparasitic bat flies. Parasite. 2020;27.

Witsenburg F, Clément L, López-Baucells A, Palmeirim J, Pavlinić I, Scaravelli D, Ševčík M, Dutoit L, Salamin N, Goudet J, Christe P. How a haemosporidian parasite of bats gets around: the genetic structure of a parasite, vector and host compared. Molecular Ecology. 2015;24(4):926-40.
